# Progress in State Medicine

**Published:** 1903-11-28

**Authors:** 


### Progress in State Medicine.

(*Concluded from page* 135.)

### HOUSING OF THE WORKING CLASSES.

Recent reports[5] presented to the London County Council show that up to March 31st, 1903, the Council had expended in capital account for dwellings for the working classes, £1,221,754 17s. 7d. The dwellings include 3,532 tenements, 384 cottages, and 324 cubicles in lodging-houses, and accommodate in all 19,628 persons. The additional accommodation which has been provided for the labouring classes, especially in the periphery of London, coupled with improved means of locomotion by electric and other trams and workmen's trains, is beginning to make its influence felt upon the congested areas of the inner zone. The new cottages at Tooting have been largely taken up by those previously resident in Lambeth, Southwark, and other crowded areas. The Newport Corporation[6] is definitely committed to a proposal for the housing of the working classes. The estimate of the committee for the scheme is:—Cost of lands, roads, and sewers, £1,000; building (66 houses at £125 each), £8,250; contingencies, £750; total (say), £10,000. They think that these houses would be eagerly taken at a rental of 3s. 6d. a week, which would mean an annual income of £600 12s., and out of this there would be the following charges:—Interest and sinking fund (about), £400; rates and taxes, £100; repairs, £30; commission for collecting rents, £15 17s. 6d.; total, £549 17s. 6d. So that if there were the full income of £600 12s., as estimated, and the outgoings did not exceed the estimate, there would be a profit each year of a fraction over £50. The class of tenants which the committee suggest should be catered for comprises men whose average wages are below 21s. a week. Though it is difficult to frame rules for the government of the houses whilst the scheme is in preparation the committee think that such rules, when made, should provide for inspection by the Corporation, to prevent uncleanliness; ejectment, without notice, of tenants who use the houses for improper or disorderly purposes, and a payment of 5s. deposit before taking possession to cover loss of keys, and repairs to windows. There will be 33 houses—that is, separate buildings from floor to roof—with two separate tenements in each, one on the ground floor and one on the first floor, approached by a separate entrance. Each tenement will consist of one living room, one bedroom, a scullery, and a w.c. at the back. The living room of the ground floor tenements will have an area of 97 square feet; on the first floor, 100 square feet; the bedroom on the ground floor, 130 square feet; and on the first floor, 100 square feet. The scullery in each case will be 7 feet 6 inches by 5 feet. There will be an open common yard to each two houses, the size of which will depend on the extent of the site, the width being about 28 feet and the length about 75 to 80 feet. The Local Government Board[7] has addressed a circular to town councils and urban district councils drawing attention to the provisions of the new Housing of the Working Classes Act, 1903. The provisions respecting borrowing powers and repayment of loans are considerably amended by Section 1 of the new Act. The effect of Sub-section 1 of this section is to extend the maximum period for the repayment of loans raised for the purposes of the principal Act, or any Acts amending it, to 80 years, leaving the actual period for repayment, subject to this limitation, to be determined as heretofore with the sanction of the board. The next subsection will prevent any loans raised for the purposes of the Housing Acts from being taken into account for the purposes of the limitations on borrowing above referred to. The Board state that they propose in future, as a general rule, to allow the full term of 80 years for the repayment of money borrowed for the purchase of freehold land, and 60 years for the repayment of money borrowed for the erection of buildings under the Housing Acts, where the circumstances are such that this may be properly done. Where money has been borrowed in recent years for these purposes, they will be ready to consider applications for sanction to the re-borrowing of the out-standing balances for 80 or 60 years (as the case may be) from the date of the original borrowing, if the money has been borrowed on terms which will admit of this. The New Act also provides for appeal to the Board by ratepayers, in the event of the local authorities doing nothing. Certain points on procedure, modification of schemes, closing orders, cost of demolition, and letting houses for the working classes, are also incorporated in the new Act.

[5] Brit. Med, Jour.. Aug. 8, 1903. [6] Pub. Health Engin., Sept. 26, 1903. [7] Brit. Med. Jour., Oct. 3, 1903.

